# Determination of Tensile Characteristics and Electrical Resistance Variation of Cables Used for Charging Electric Vehicles

**DOI:** 10.3390/polym17101317

**Published:** 2025-05-12

**Authors:** Elena Roxana Cosau, Viorel Goanta, Igor Blanari, Layth Alkisswani, Fayez Samara

**Affiliations:** Mechanical Engineering, Mechatronics and Robotics Department, Mechanical Engineering Faculty, Gheorghe Asachi Technical University of Iasi, 700050 Iasi, Romania; elena-roxana.cosau@student.tuiasi.ro (E.R.C.); igor.blanari@academic.tuiasi.ro (I.B.); layth.alkisswani@student.tuiasi.ro (L.A.); fayez.samara@student.tuiasi.ro (F.S.)

**Keywords:** charging cable, polymer coating, tensile device, electrical resistance

## Abstract

In this paper, the tensile behavior of the power cable used for charging electric machines was analyzed. It is known that such a cable, consisting of several conductors, polymeric sheaths and textile core wire, can be subjected to mechanical and thermal stresses that lead to failure to operate at the desired parameters or to total interruption of operation. The mechanical stresses to which the cable is subjected are, in general, bending and tensile stresses, with the development of normal stresses, torsional stresses where tangential stresses occur and, possibly, shock stresses produced by several causes. The present paper proposes to determine some mechanical characteristics of the mentioned conductors resulting from tensile stress for testing, using a special device built for this purpose. In order to obtain other mechanical characteristics also, a finite element analysis has been carried out, the results of which are compared with those obtained from the experiment. Another type of determination was also carried out using the tensile device: the variation of the electrical resistance of one of the electrical conductors of the cable during tensile stress was recorded.

## 1. Introduction

In general, in service, power cables are not subject to high mechanical stress or excessive temperature variations. There is, however, a category of cables, such as those mounted on poles outdoors, that is subject to stresses due to their own weight, ice deposition and wind gusts and temperature variations. Cables used for charging electrical machines may be subject to various mechanical stresses and environmental stresses due to their specific use. Thus, tensile stresses due to accidental handling, bending stresses or torsional stresses may occur. The charging performance of electric vehicles (EVs) is closely related to the mechanical stress behavior of the charging cable. From this point of view, the requirements for charging cables for electric vehicles are more stringent than for traditional cables. These requirements must ensure the reliability of the cable and the safety of charging in the shortest possible time.

In the context of the increasing number of electric vehicles and the requirement for appropriate charging infrastructure, electric cables intended for this purpose must meet technical safety and performance standards. Mechanical tests are essential to evaluate the resistance of cables to physical damage and frequent use conditions [1]. The main purpose of mechanical testing is to evaluate the resistance of cables to mechanical stresses such as bending, torsion, abrasion, shearing, wear of the polymer outer sheath or other stresses that may occur during handling, transportation and the use of cables for charging electric vehicles [2,3,4]. Standards in the field of electric vehicle charging cables consider a number of issues such as the following:

- Common test methods for insulating and sheathing materials of electric cables and optical cables: Part 202: General tests—Measurement of thickness of non-metallic sheath (IEC 60811-202, [5]);
- Power cables with extruded insulation and their accessories (IEC 60502-1, [6]);
- Tests on electrical cables and optical fiber cables under fire conditions (IEC 60332-1-2, [7]);
- Charging cables for electric vehicles for rated voltages up to and including 0.6/1 kV: Part 1: general requirements (IEC 62893-1, [8]);
- Electric cables: charging cables for electric vehicles (EN 50520:2020/A1;2021, [9]);
- Electric and optical fiber cables: test methods for non-metallic materials (EN 60811-100, [10]).

From the study of standards and scientific papers on cables used for electric vehicle (EV) charging, it appears that there is not much research dealing with mechanical testing and the determination of mechanical strength under various stresses. In a study by Singh et al. [11], a comprehensive overview of different technologies for the charging of EVs is given. The charging infrastructure is presented, highlighting the charging modes, charging methods, electrical energy storage technology and standardization and integration of charging sources and charging cables, with discussions about the materials and design of charging cables. There is no discussion about the correlation between the chosen material and the defects that may occur during operation. Lin et al. [12] present a finite element analysis of a finished product with respect to an EV charging cable. The results of this research consist of some mechanical characteristics provided from the analysis. Unlike the study conducted in this paper, the cable is not presented as an assembly formed by spiral wires. The study presented by Lin et al. in [13] discusses the ways of testing as well as how to design some devices that should be used in the mechanical testing of these types of cables. One shortcoming of these tests seems to be the test mode related to the cable gripping device. The paper mentions the negative aspects that can occur in such a stress, i.e., “slip” and “end break” phenomena. In order to mitigate the effects of breakage or uncontrolled damage during tensile tests, a statistical calculation was performed. Jin et al. [14] present the Mixed-Integer Linear Programming model that provides, in real time, a simple heuristic algorithm to link the EV under load to the charging station via a charging cable. In addition, a communication protocol between the charging station and the power grid is presented for real-time energy storage planning. Under these conditions, the charging cable is given additional communication functions between the vehicle regarding the required charging capacity and the availability of the electrical grid. On the other hand, quite a number of problems arise when cables, wires, wire conductors, etc., are subjected to tensile stress. Depending on the device used, these problems are related to the crushing that occurs in the gripping area, slipping or crushing on the rollers and the asymmetry of the test. For this reason, a new gripping device for tensile stressing of cables was proposed by Xiao et al. in [15]. A finite element analysis of this device was also carried out in order to verify the feasibility of the device used. However, as seen from the paper, there are still some problems to be solved in the spring-guided gripping mechanism. Stevens et al. [16] present some fatigue tests on a model hydrogenated nitrile butadiene rubber (CC-HNBR) composite reinforced with a single carbon bead. The tests were performed using conventional grips, which is an initial deficiency, in relation to the device presented in this paper, which was designed and made by the authors. In any case, here, the aim was more to destroy the sample over time by using a thermal imaging camera and assimilating infrared images with irreversible damage. Jingle Jabha et al. [17] investigated conductor failure via capacitance damage based on a non-destructive method, by which the resistivity and stiffness are detected and monitored. In analogy with various electrical properties, the dielectric strengths of different cross-linked polyethylene (XLPE) cables were analyzed. The results explain that by developing an acoustic emission (AE) technique, the performance degradation of power cables can be evaluated, and the exact location of the initial damage during tensile stress can be identified. It is also observed that before failure, these cables have the ability to slip within a range of 150% to 200%. There is research that presents tests of wires or cables at elevated temperatures [18,19,20,21,22,23]. Kuzmanov et al. [18] presented a way of determining some of the mechanical properties of wires subjected to tensile tests. Tensile tests of Inconel 600 wire were performed at 700 °C, 800 °C and 900 °C. The ultimate strength, yield strength and percent elongation after breakage were determined. Anderson et al. [19] simulated heating based on finite element analysis. Here, two models were developed and presented: an empirical one, with unsatisfactory results from our point of view, and an analytical one. Based on the experimental results taken from the literature, an attempt was made to validate these models, with different working parameters considered. In the study presented by Rickman et al. [22], the possibility of degradation of mechanical characteristics under the existence of heating–cooling cycles was studied. As a result of the degradation thus observed, it was found that there is a close correlation between the mechanical properties of a conductor and the different phases of mechanical degradation. An important study here was to verify whether there is a correlation between deterioration, mechanical perforations and the twisted design of the cable. Zichuan et al. [23] presented the variation of the performance of some conductors used in the nuclear industry based on temperature variations by sudden cooling–heating.

In this paper, two types of cables used for charging electric vehicles were subjected to tensile stress. On this basis, the mechanical characteristics resulting from this type of test were determined. It should be mentioned that for the tensile test, a special device attachable to a universal testing machine was conceived, designed and realized. This device, in comparison with similar ones used for the same purpose, offers several advantages and novelties, which will be emphasized in the text following. Also, on the basis of axial loading, the electrical resistance was measured for one of the component cables of the assembly, following its variation over time and in relation to the applied force. As will be seen in the paper, interesting and perhaps unexpected dependencies are obtained both in the variation of electrical resistance over time and in its dependence on the test force.

## 2. Materials and Methods

### 2.1. Materials Used for Static Tensile Testing

**Figure 1:**
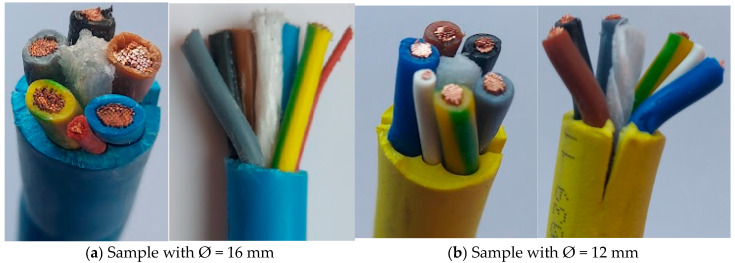
Samples used in tensile testing. Ø is cable diameter.

Two types of cables used for charging electric vehicles were used in the tensile tests, both of which are of flexibility class 6 according to IEC 60228 [24]. The first of these (blue) has an outer diameter of 16 mm, with 6 inner cables and a central textile wire (Figure 1a). The second cable tested (yellow) has an outer diameter of 12 mm, 6 inner cables and an inner textile wire (Figure 1b).

The electrical conductors are made of very fine copper wire, with a specific mass of 72 kg/km. The conductor sheath is made of PVC TI2, and the outer sheath is made of PVC TM2, according to DIN VDE 0281 part 1 + HD 21.1. The tensile samples were cut from industrial cables that were to be equipped with the connection plugs.

### 2.2. Description of the Cable Configuration and Materials Introduced in the Finite Element Analysis

**Figure 2:**
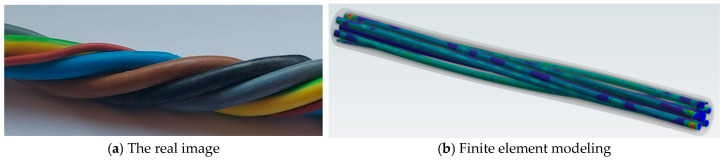
Bundle of cables in strand form.

The material models used in the finite element analysis are shown in Table 1 below. These include the mechanical properties of the copper wires, the inner and outer protective sheathing, generically referred to as PVC, the textile yarn inside the assembly and the metal material from which the rollers are made. These properties make it possible to model the structural behavior of the cable components by specifying mechanical parameters such as Young’s modulus, Poisson’s ratio, the tangential modulus, yield strength and others. The observation is made here that the cables in the interior of the outer propylene sheath are formed in the shape of a strand, i.e., they are twisted together in the same direction (Figure 2a). The finite element analysis was performed with the inner wires arranged in this configuration (Figure 2b). It is mentioned that in Figure 2b, the inner cables have already been loading.

**Figure 3:**
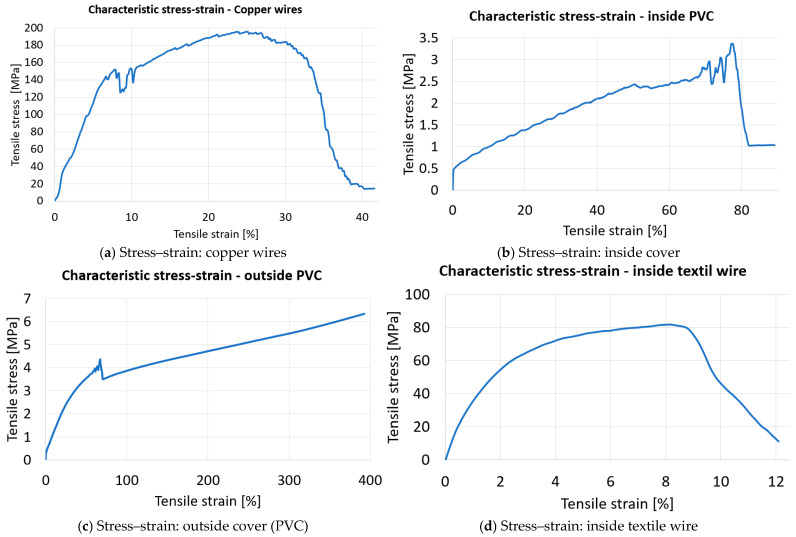
Stress-strain characteristic curves for four types of materials.

Separately, the following components were also subjected to tensile loading with the same device as previously used: the outer sheath, one of the inner cable sheaths, the copper wires of an inner cable and the central textile yarn. In view of the facility provided by the device, the metal rollers on which the cables are wound were moved and subsequently fixed so that the cables/wires of different diameters lay with the axis in the direction of the actuating force. Since only a single material is involved for each component, specific stress–strain characteristic curves were plotted, from which the data required for the finite element analysis process were taken. These curves are shown in Figure 3. The characteristics of the materials used for each component in the pair are those given in Table 1.

### 2.3. Method and Device Used for Tensile Tests

**Figure 4:**
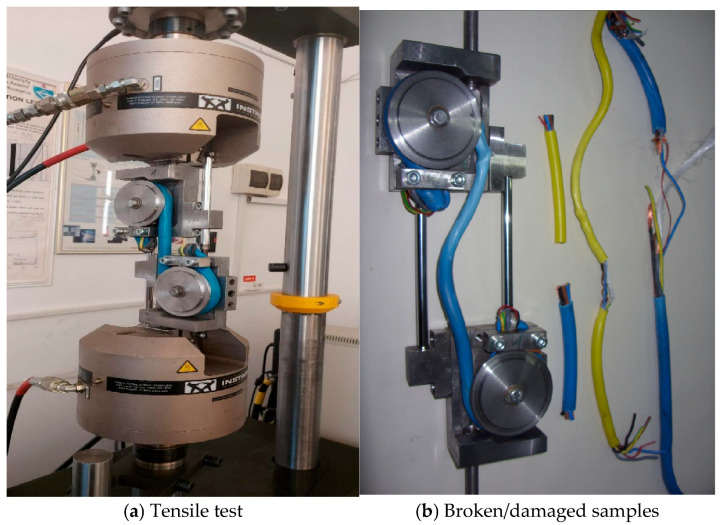
Clamping the device in the testing machine and broken/damaged samples.

The tensile tests were performed using a Instron 8801 universal testing machine (825 University Ave. Norwood, MA, USA) on which a purpose-built device was mounted (Figure 4a). The device used for tensile testing has several characteristics, as described below. The device is composed of two similar parts (semi-devices) but mounted asymmetrically in the test machine’s grips. Each part (semi-device) contains a wheel around which the cables are wrapped and clamped in two grips/clamps (Figure 4b). The cables/wires pass over the cylinder sleeves because of the need to not be crushed by the clamping in the test machine’s grips. If the tests were performed based on direct clamping of the cables, crushing stresses would occur in the clamping area, leading to the cable breaking in the grips. Under these conditions, the test could not be validated. As a result, the transition from the straight stress zone to the reaction zone should be as smooth as possible, without significant stress on the cable/wire. After passing over the roller surface, the ends of the cables are clamped in the flanges with a squeeze grip, but the tensile force here is greatly diminished, given the previous frictional force with the rollers.

## 3. Results Obtained from the Static Tensile Test of Cables

### 3.1. Advantages of Using the Universal Testing Machine Attachment Device

The cable testing device has the following advantages:

- It is provided with a system of transverse guidance of the rollers so that cables of different diameters can be tested while maintaining axiality between the geometrical axis of the cables and that of the testing machine. For this purpose, the rollers over which the wire is passed are movable in such a way that the geometrical axis of the wire between the two rollers always corresponds to the stress axis on which the equal and opposite forces applied from the testing machine are arranged. The displacement of the rollers shall be affected by means of screws and guided on the rectangular groove in each of the base plates.
- On the outside of the rollers, a circular polyamide cover has been fitted, which can be easily replaced. This sleeve has a certain radius to the outside; therefore, it will not have a linear generator. Under these conditions the squeezing effect of the cable by the metal cylinder at the contact between the two components— the cylinder and cable—will be reduced. Depending on the diameter of the cable, the polyamide cover wheels with the corresponding radius are changed.
- The test system contains two metal guide rods between the upper and lower (semi-device) parts of the device in order to balance the moments that lead to the rotation of one or the other part (Figure 4b). The rods rest on two cylindrical guides, one for each semi-device, designed so that the moments developed from the asymmetry of forces in each semi-device cancel each other out, given their opposite direction. If these rods did not exist, the rotations arising from the eccentricity of the two forces on the load cylinders would result in a rotational moment about the load axis for both the upper and lower parts of the device. These moments should be taken over by the testing machine, which is not convenient. By mounting the two guide rods, the rotating moments from the two semi-devices cancel each other, having different directions.

### 3.2. Results Obtained from the Static Tensile Test

**Figure 5:**
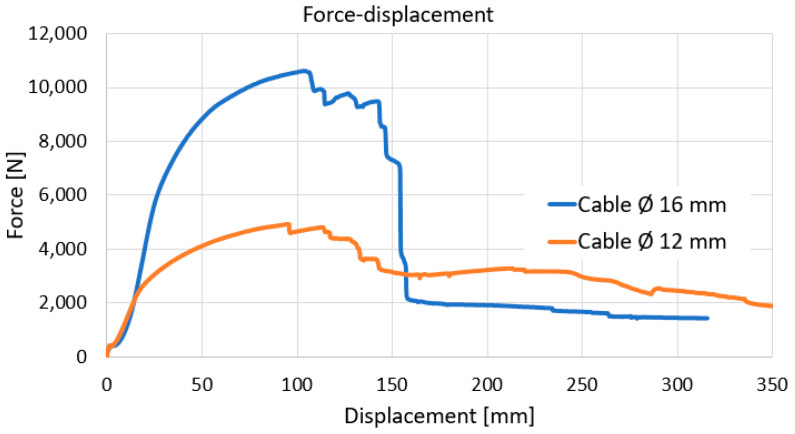
Force-displacement curve (F-Δl) for cable with total diameter of 16 mm.

By static tensile loading, several characteristic results are obtained, as well as force–displacement curves [25]. The force-displacement curves for the 16 mm and 12 mm total diameter cables are shown in Figure 5. It is noted that the cables are twisted internally in a strand shape. For the curve representing the cable with an outer diameter of 16 mm, several characteristics can be observed, as described below.

It is observed that at the beginning, there is an increase in the slip with a small increase in the force due to the tightening of the cable on the rollers and fixing in the grips. After the tightening zone of the cable at the beginning, it is observed that there is an almost linear zone signifying the elastic zone, i.e., at the cessation of force, the cable will return to its original shape and dimensions. After the elastic zone, the variation in F-Δl curves occurs; it is not known how much of the curvature is due to the copper conductors and polyvinyl sheaths entering the plastic deformation zone and/or the cables inside the cable returning from stranding. After this curved zone, a sudden force drop occurs repeatedly, which denotes failures of the inner PVC sheathing (see individual characteristics in Figure 5). At these times, no failures of the outer polyvinyl sheathing are observed. As a result, it is expected that the copper conductors also do not fail before the polyvinyl inner sheaths.

It should be noted that since we are dealing with a set of different cables and materials, we cannot consider a specific stress–strain characteristic curve. The force–linear displacement curve for the cable with a total diameter of 12 mm is shown also in Figure 5. It can be seen that the shape of this curve is similar to the one for the cable with a total diameter of 16 mm. At the beginning, the cable is better stretched on the rollers and fastened in the grips. Once the maximum force of about 5 kN is reached, the inner polyvinyl sheaths fail one by one, leading to a continuous decrease in force. The elongation is primarily due to the deformation of the polyvinyl shells. However, here, there is no sudden drop in force but rather a continuous one based on the successive failures of the cables.

### 3.3. Results Obtained from Finite Element Analysis of Tensile Loading of 16 mm Outer Diameter Cable

The 16 mm diameter cable was subjected to finite element analysis by tensile stressing it by simulating the test on the roller device described above. Previously, several tensile tests were carried out, observing the modes of deformation and rupture. With FEA Ansys software (version 2022 R2), an attempt is made to model the modes of deformation and fracture in order to approximate the two approaches in terms of the results obtained. This was carried out in order to further use the finite element analysis, calibrated on the basis of previous experimental results and also under other stress conditions (at different temperatures, at different test speeds, etc.). The finite element analysis process is carried out in several steps, which will be described in the text following. In addition, as shown above, having to test an assembly of cables and materials, it is not possible to determine from the experiment the stresses in the components and, especially, the maximum stress [26].

**Figure 6:**
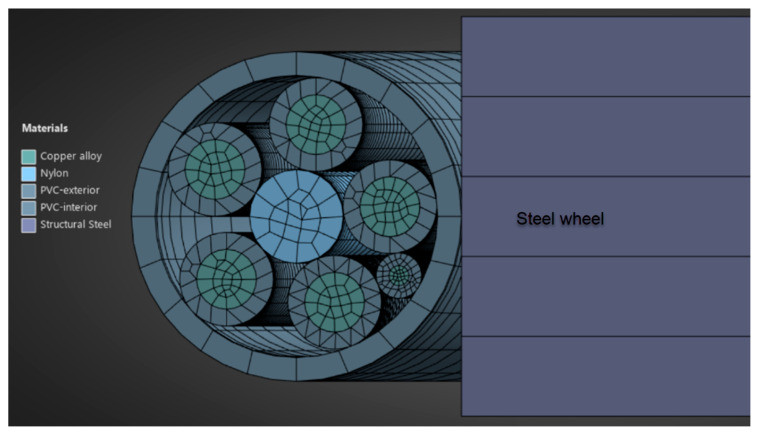
Cable component discretization mode.

Figure 6 shows the discretization of the inner components and the outer casing of the assembly used in the mechanical tests. The drawing also shows part of the steel wheel on which the cable is wound. For the inner conductors, the center part is the copper wires, and the outer part is the polypropylene cover. Lengthwise, the inner cables are stranded, as shown in Figure 2.

**Figure 7:**
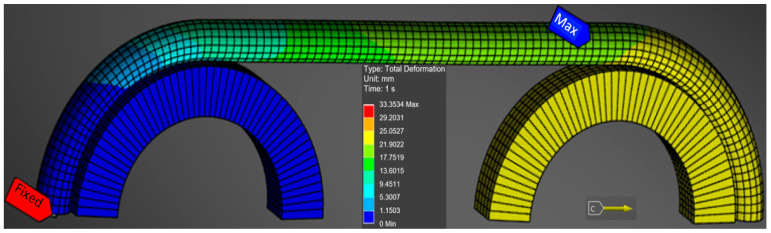
Modeling the cable test mode in FEA.

Figure 7 shows how the loading is realized in finite element analysis. The left wheel is fixed, while the right wheel is movable. In this way, a tensile force is introduced into the cable, similar to that in the previously presented experiment. Figure 7 also shows the variation of the displacements. It is noted that the maximum displacement of 33.35 mm is recorded at one of the inner cables.

**Figure 8:**
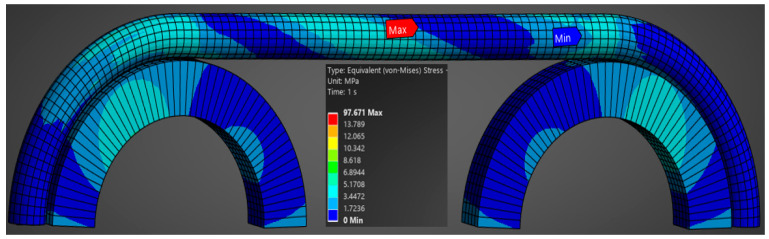
Von Mises tension map.

It should be noted that the finite element analysis was not carried out up to the rupture zone but only until shortly after the yield limit was reached. Under these conditions, Figure 8 shows the global von Mises equivalent stress map for the whole assembly.

**Figure 9:**
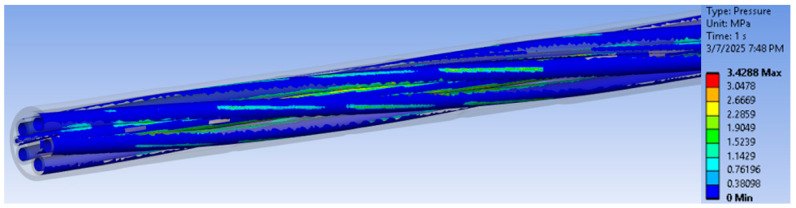
Map of pressures between cables at maximum force.

The advantage of such a finite element analysis of an assembly containing several components is that it can deliver the equivalent stresses, and we can determine the location of the maximum stress values, which cannot be calculated analytically or experimentally. In the case of the experimental tensile stress to failure test, we cannot set up a specific stress–strain characteristic curve, since we are dealing with an assembly of components made of different materials. For this reason, in the previous chapter, only the force–displacement curves were presented, with both quantities being taken globally from the testing machine. By introducing the material characteristics into the finite element analysis, one can see the stress map and, as a consequence, the location of the maximum stress. As can be seen from Figure 9, the location of the maximum stresses is in the section of initial contact with the metal wheel but somewhere on a cable inside. On the other hand, also from Figure 9, it can be observed that the rotation of the colors represents the same value of the stresses. This happens as a result of the initial rotation of the inner strand-like cables and un-rotation under the action of tensile force. Figure 9 shows a map of the pressures occurring at the contact between the inner cables as a result of loading and un-rotation during the tensile test. The pressures do not appear to have large values but are sufficient to provide a significant frictional force between the cables.

**Figure 10:**
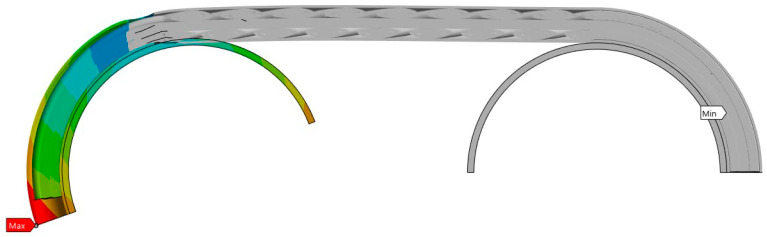
The pattern of breakage initiation, in longitudinal section.

Large displacements of the moving wheel can lead to breakage, which, as can be seen in Figure 10, also occurs in the initial contact area with the wheel.

### 3.4. Results Obtained from Tensile Test, Recording the Change in Electrical Resistance of a Single Conductor

#### 3.4.1. Electrical Circuit Bonding Mode

**Figure 11:**
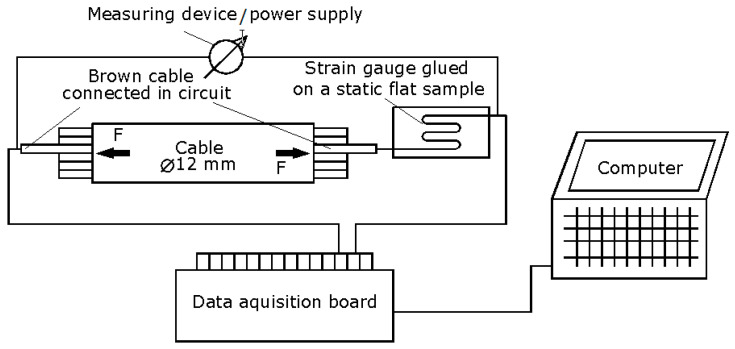
Electrical connection scheme for data acquisition.

During their use, cables can be subjected to various stresses such as bending (tension + compression), twisting, shock, etc. In the event of a tensile stress exceeding certain limits, a change in the electrical resistance of the conductors is possible, which may affect the load-carrying capacity of the whole assembly. For these reasons, in the course of this sub-chapter, a tensile test has been carried out on a cable as a whole, as was conducted previously, and a single cable out of all the five existing cables has been connected in an electrical circuit of the form shown in Figure 11.

The electrical circuit in Figure 11 contains the following components:

- A cable with an outer diameter of 12 mm, having six inner electrical wires/conductors, five of which are approx. 3.2 mm in diameter and the sixth approx. 1.7 mm in diameter, and a textile wire;
- An aluminum plate, to which a 120-ohm resistance tensiometer strain gauge has been glued;
- A measuring device with a dual function: to check the initial resistance and to feed the electrical circuit to measure the resistance;
- A National Instruments NI-USB-6009 data acquisition board;
- A laptop on which Labview software (NI Labview, version 19, 64 bit), used for data acquisition, is installed.

From the main assembly of yellow outer color, the brown cable was chosen to monitor the variation of the electrical resistance when the whole assembly is subjected to tensile stress.

The yellow assembly-type cable will be subjected to tensile stress while the component cable is wired in electrical circuit for resistance variation monitoring. It is energized by the meter, while the connecting ends are connected to the data acquisition board.

An acquisition program scheme is used for data acquisition that has been designed and developed within the Labview program. The signal input to the DAQ Assistant is displayed on a graph, and the data are transferred to a text file (write to measurement file). Since the data acquisition rate from the test machine is 1 s, this value was also chosen in the DAQ Assistant. This setting was made in order to subsequently superimpose the variation of the force versus the variation of the electrical resistance of the cable.

For data acquisition in terms of electrical resistance, Labview software (NI Labview, version 19, 64 bit) requires that the electrical resistance should vary between 100 ohms and 1 KΩ. While the upper value can be changed, the lower value cannot be less than 100 ohms. This is the reason for the introduction of the series of strain gauges, which has a resistance of 120 ohms. It should be noted that the pad to which the strain gauge was bonded was not stressed; therefore, there was no change in its resistance during the test. Consequently, the variation in electrical resistance occurred only as a result of the tensile stress of the brown-colored conductor, bonded in the electrical circuit shown above.

#### 3.4.2. Mechanical Clamping and Electrical Connections

**Figure 12:**
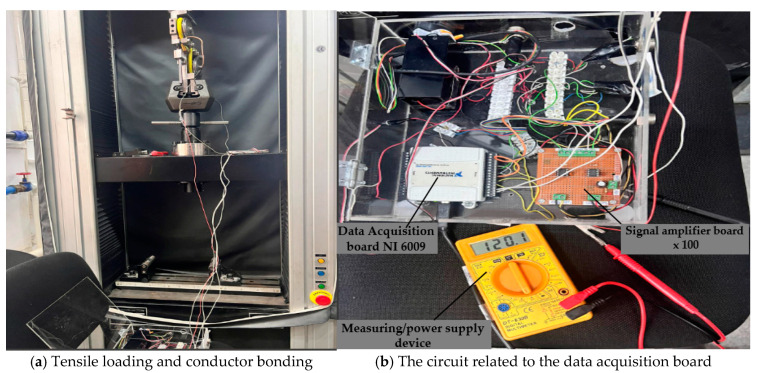
Conducting tensile tests with monitoring and data acquisition of the electrical resistance of the brown cable.

Figure 12 provides images of the assembly-type cable tensile test procedure on the testing machine. Figure 12a shows the cable mounting in the previously used and described device, as well as the device mounting on the universal testing machine. Figure 12b shows the cable connection to the data acquisition board during the tests.

It is taken into account not to intervene on the electrical connections during tensile stress. A temperature of 21 degrees Celsius was maintained constantly throughout the test.

#### 3.4.3. Experimental Data Processing and Conclusions Drawn from the Acquisition of Electrical Resistance Variation

**Figure 13:**
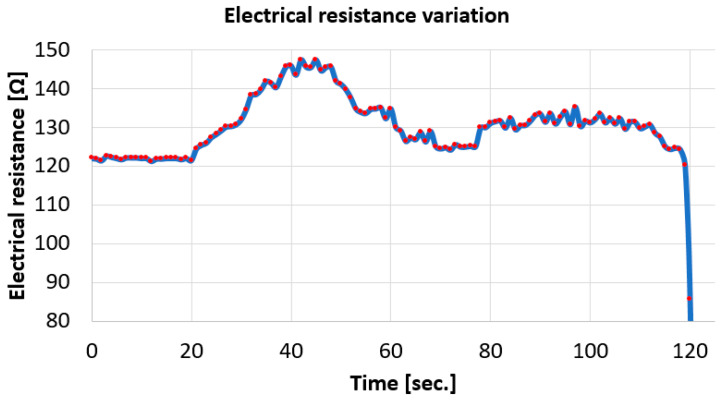
Variation in resistance of brown cable/conductor under tensile stress.

Based on the data acquisition, regarding the variation of the electrical resistance of the brown conductor during the tensile test, the graph in Figure 13 was drawn, which shows this variation over time, with a sampling rate of 1 s, as previously mentioned.

From the variation analysis in Figure 13, the following can be observed:

- Initially, the total resistance of the monitored circuit has a value of approx. 121 ohms, given that the resistance of the strain gauge used in the circuit is 120 ohms;
- The initial area with constant resistance at approx. 121 ohms is before the start of the tensile test, given that data acquisition was started first, and then the tensile test was started (after approx. 20 s);
- At the beginning of the test, an increase in resistance is observed: the largest increase was approx. 28 ohms.

The equation for the electrical resistance R is:

(1) $$R=\rho \frac{l}{S}$$

where *ρ* is the electrical resistivity of the conductive material, l is the total length of the conductor, and S is the cross-sectional area of the electrical conductor.

It is obvious that at the moment the test began, given that the length l increases and the cross-sectional area decreases, the resistance value increases significantly:

- After approx. 45 s, although the tensile test continues, the resistance begins to decrease. This happens because the thin copper conductors that make up the brown conductor begin to break. In fact, sudden drops in resistance are also observed before, caused by the failure of the copper conductors.
- Between the times of 60 s and approx. 120 s, the value of the electrical resistance varies less, with temporary increases and decreases. This is attributed to the achievement of a relative balance between the increase in resistance produced by the elongation of the thin copper conductors and the breakage of some of them. In this interval, two levels can be observed: between 62 and 77 s and between 78 and 113 s. Between these, respectively, between seconds 77 and 78, the resistance increases as a result of the fact that the stretching of the copper conductors occurs without the breakage of other conductors.
- After approx. 120 s from the beginning of the acquisition (approx. 97 s from the beginning of the test, all the thin copper conductors within the brown conductor break.

**Figure 14:**
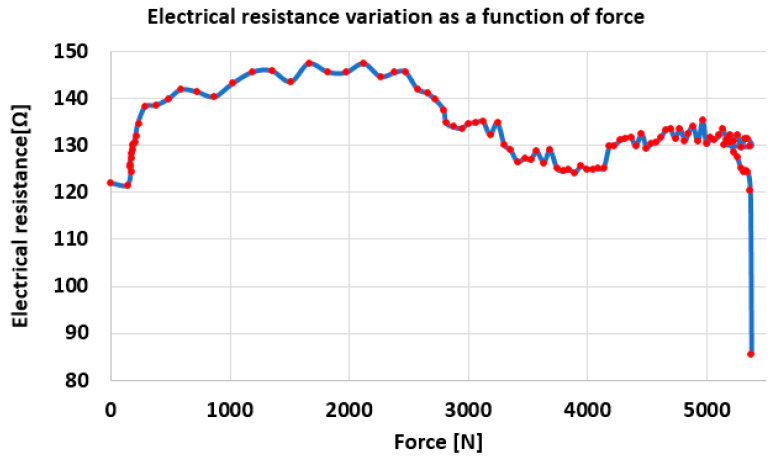
Variation of the electrical resistance of the brown conductor in relation to the tensile stress force.

In Figure 14, the variation in the electrical resistance of the brown conductor in relation to the tensile stress force is plotted.

From Figure 14, it can be seen that at first, as the force increases, the resistance value also increases. At an increase in the force of approx. 2000 N, the resistance value increases by approx. 28 ohms. This increase is due to both the increase in the length of the thin copper conductors and the decrease in their cross-section, under the action of the stress force (see Equation (1)). With this, small decreases in the electrical resistance are also observed, most likely caused by the breakage of one copper conductor from the bundle of conductors that make up the brown cable. Starting from the force value of 2470 N, a decrease in the electrical resistance occurs. This decrease is caused by the breakage of more and more copper conductors. Although the elongation continues, breaking a large number of copper conductors, an overall decrease in the electrical resistance occurs. Moreover, from Figure 4 it can be seen that after a force value of approx. 2500 N, the slope of the force variation curve in relation to the elongation changes, respectively, decreases. This denotes the entry into the plastic deformation zone. Of course, plastic deformation can also occur in the cable sheaths, in addition to the outer sheath. However, the copper conductors also enter plastic deformation early, which leads to a decrease in electrical resistance in the interval in Figure 14, as shown above. After the force of 4000 N, a new increase in resistance is observed, which implies that the decrease given by the breakage of the copper conductors is smaller than the increase in resistance based on its elongation. After the force of approx. 5200 N, when a decrease in force also occurs, the electrical resistance also decreases due to multiple breakages of the copper conductors.

## 4. Conclusions

In general, electrical cables are not tested for mechanical characteristics. Their design is based on the required electrical characteristics. However, electric vehicle charging cables are subjected to significant mechanical loads, which makes tests for mechanical characteristics as well as integrity and load capacity maintenance very important. This is also the reason why in this paper, certain mechanical characteristics of the cables subjected to static tensile testing, used for electric charging of motor vehicles, were determined. In general, there are few records in the specialized literature regarding the determination of the mechanical characteristics of these types of cables. The tensile test was performed using a device specially built for this purpose.

The tensile test of the two types of cable highlighted a behavior that also takes into account the possibility of unwinding/rotating the component cables from the strand inside the outer sheath. A greater influence is observed in the characteristic curves of the component cables’ outer sheaths than of the copper conductors, highlighted by the greater curvature at the beginning of the force–displacement curve. Although the two cables used in the static tensile test have the same configuration, of course, having different diameters, it is found that the 16 mm diameter cable shows a sudden drop in strength, which means an almost simultaneous failure of the inner components and the outer sheaths.

It was found that in the case of testing on the testing machine, we cannot talk about stresses because we have an assembly of cables, electrical conductors, etc. That is why finite element analysis was useful in order to observe the areas of maximum stress and to take into account the friction of the cables.

Regarding the variation in the electrical resistance of one of the conductors during the tensile test, it is observed that although a significant increase in the length of the copper wires is obtained, which could also lead to an increase in the electrical resistance, from a certain point on, the electrical resistance begins to decrease. This happens as a result of the fact that the copper conductors begin to break, one after another, and the increase in electrical resistance through elongation is diminished by its decrease through the breaking of the conductors.

## Figures and Tables

**Table 1:** Material characteristics used in elemental analysis.

	Young’s Modulus	Poisson’s Ratio	Yield Strength	Tangential Modulus
Material	[MPa]		[MPa]	[MPa]
Copper	110,000	0.34	150	1150
Outside cover	1100	0.42	4	0
Inside cover	1100	0.42	2.5	0
Textile wire	3500	0.39	50	0
Steel	2.1 × 10^5^	0.3	360	4400

## Data Availability

The original contributions presented in this study are included in the article. Further inquiries can be directed to the corresponding author.
